# Incorporation of Greater Palatal Artery Pathway Projection into a Static Surgical Guide

**DOI:** 10.3390/dj13040152

**Published:** 2025-03-30

**Authors:** Alexandru E. Petre, Andrei Macris, Cezar Ionita, Gabriel Cojocariu, Sergiu Drafta

**Affiliations:** 1 Prosthetic Department, Faculty of Stomatology, “Carol Davila” University of Medicine and Pharmacy, 020021 Bucharest, Romania; alexandru.petre@umfcd.ro (A.E.P.); cezar.ionita@drd.umfcd.ro (C.I.); sergiu.drafta@umfcd.ro (S.D.); 2 Private Practice, 011536 Bucharest, Romania; gabriel.cojocariu@dentville.ro

**Keywords:** the greater palatal artery pathway, free gingival graft, connective-tissue graft, palatal mucosa, digital surgical guide

## Abstract

**Background/Objectives:** The purpose of this study was to develop a digital workflow to incorporate the mucosal projection of the pathways of the greater palatal artery into a static surgical guide used for free gingival graft harvesting and connective-tissue grafting techniques. **Methods:** A cone-beam computed tomography file was uploaded and segmented using specific tools from digital design software. The artery pathways were identified and marked on cone-beam computed tomography. A standard tessellation file format was obtained and uploaded into three-dimensional mesh-processing software; this was merged into an intraoral scan file. New files were obtained and uploaded into three-dimensional modeling software. The final model with projections of the artery pathways was generated using specific tools. The digital model was uploaded into guided surgery planning software to design a digital surgical guide that could later be printed with the artery pathways marked on its surface. **Results:** The static surgical guide to the palatal mucosa could be used during a surgical approach for marking the safe-zone area against the artery pathways. **Conclusions:** The proposed technique is a viable method for visualization and marking the artery pathway projection on a static surgical guide when performing free gingival graft harvesting and connective-tissue grafting techniques.

## 1. Introduction

Free gingival grafts (FGGs) and connective-tissue grafts (CTGs) are frequently used in periodontal surgery and implantology for various purposes, including for gingival recession treatment and root coverage [1], increasing gingival thickness and keratinized tissue [2], ridge augmentation [3], vestibuloplasty [4], papilla reconstruction [5], and improving esthetic results for patients with immediate implants [6].

FGGs and CTGs are typically harvested from the palatal mucosa and the maxillary tuberosity areas due to their histological similarity to the gingival tissue [7,8]. The palatal mucosa is the most frequently harvested area due to the generous blood-supply vessels and superior quality of the grafts compared with tuberosity-free grafts which often exhibit a tendency toward hyperplasia [9]. Several techniques have been described for harvesting FGGs from the palatal mucosa: the trapdoor approach [10]; the use of one horizontal and two vertical incisions [11], two crescent-shaped incisions [12], two horizontal and two small vertical incisions [13], or a single horizontal incision [14] to harvest subepithelial connective tissue grafts; and the de-epithelialized gingival graft technique [15]. Regardless of the technique used, one of the most common complications is injury to the greater palatal artery or the palatal nerves [16], leading to hemorrhage or paresthesia. Detailed knowledge of the soft-tissue anatomy is necessary to reduce the risk of complications [17], and different types of pre-surgical approaches to artery suturing have been proposed [18].

The greater palatal artery enters the area of the hard palate alongside the greater palatal nerve through the greater palatal foramen. It then extends anteriorly through a bone groove at the beginning to the incisive canal, where it anastomoses with the nasopalatine branch of the sphenopalatine artery [19]. It comprises three branches: canine, medial, and lateral. Four types of greater palatal artery branches have been described, depending on the origin of the medial and canine branches [20]. The main branch of the greater palatal artery measures 1.2 mm in diameter in the molar region, decreasing to 0.8 mm in the canine region [21].

The greater palatal artery has been extensively studied, and its course and variations have been documented. Different authors described a safe zone for harvesting in a systematic review [22]. The authors calculated the distance from the greater palatal artery to the cementoenamel junction (CEJ) of maxillary teeth. They also proposed the delimitation of the safety zone by subtracting the standard deviation and 2 mm of space from the corresponding biological width of the CEJ. In a study on cadavers [23], it was demonstrated that the greater palatal artery is located deeper in those with a deep palatal vault, and the distance between the CEJ of maxillary teeth and the greater palatal artery is greater for those with a deep palatal vault compared with flat palatal vault cases. According to a different study [21], the safest zone for harvesting is the second premolar area, which has a thickness of 4 mm and a width of 9.3 mm.

Techniques such as ultrasound [24], magnetic resonance imaging (MRI) [25], and cone-beam computed tomography (CBCT) have been proposed for identifying the path of the greater palatal artery, with CBCT being particularly useful due to its ability to visualize bony palatal grooves [26].

To minimize the risk of complications, digital surgical guides [27] have been designed to aid in graft harvesting. The incorporation of guided digital techniques can increase the precision, predictability, and safety of the treatment [28].

Exact prevalence data on greater palatal artery injuries during palatal soft-tissue graft harvesting are not extensively documented in the literature. However, such injuries are recognized as potential complications, primarily due to the anatomical course and variations of the greater palatal artery. To minimize the risk of greater palatal artery injury, it is essential for clinicians to have a thorough understanding of palatal anatomy and to adhere to established safety guidelines during graft harvesting procedures [22].

The present paper describes a digital technique used to obtain a surgical guide containing the projection of greater palatal artery pathways on the palatal mucosa to aid in determining the depth and breadth of FGGs. The technique is based on knowledge of greater palatal artery anatomical landmarks and pathways, the safe zone [22], and CBCT three-dimensional (3D) reconstruction.

## 2. Materials and Methods

**Figure 1:**
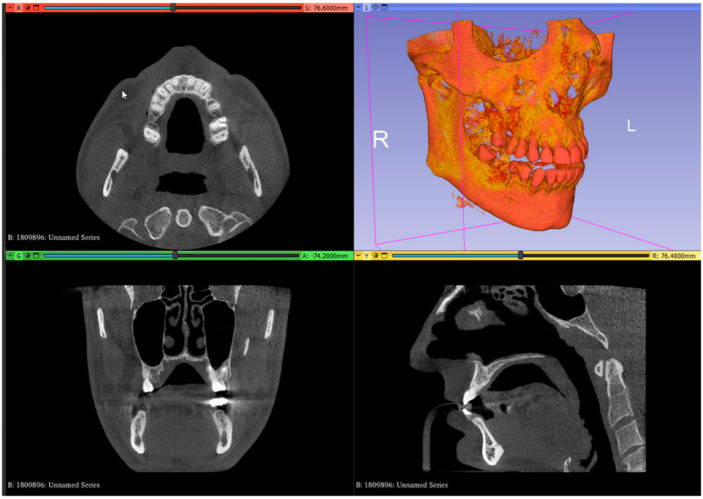
Uploaded CBCT scan in the analysis and scientific visualization software.

**Figure 2:**
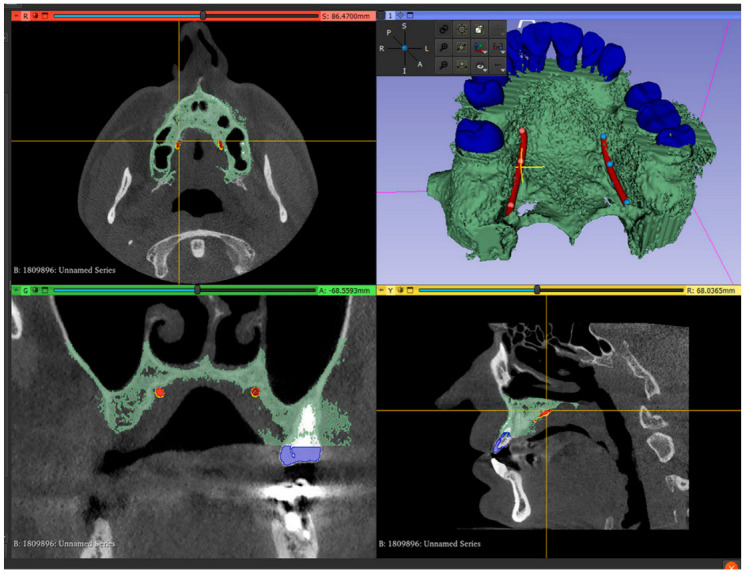
Segmented model and IOS model superimposed, with both greater palatal arteries pathways marked.

**Figure 3:**
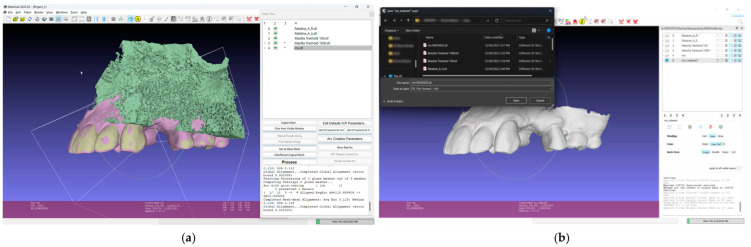
Mesh processing: (**a**) All segmented models and the IOS superimposed in the mesh-processing software; (**b**) STL format of the IOS saved with system coordinates of the obtained models.

**Figure 4:**
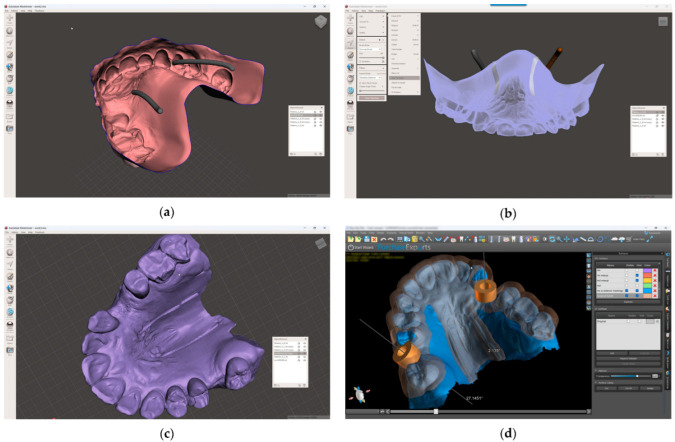
Model alignment and guided surgery planning: (**a**) The IOS and the greater palatal artery models uploaded into 3D-modeling software; (**b**) All four models and the IOS aligned; (**c**) Model with mucosal projection of the greater palatal artery pathways after Boolean operations applied; (**d**) Digital surgery guide for the FGG and the implant surgery guide combined.

**Figure 5:**
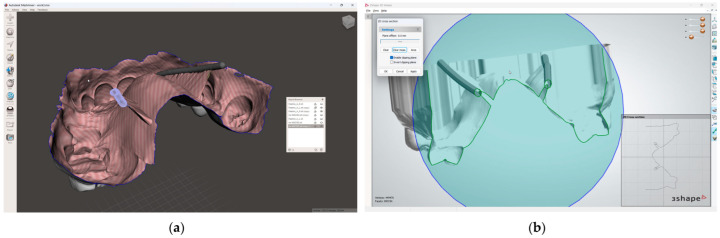
Greater palatal artery pathway visualization: (**a**) IOS with Boolean operations applied for 3D-model design; (**b**) 3D-model design with surgical harvest area visualization.

The technique involves using various software tools for free image analysis and scientific visualization, 3D mesh processing, 3D modeling, and guided surgery planning, along with known distances from the greater palatal artery to the CEJ of maxillary teeth described in the literature, with values between 13.9 ± 1 mm [22] and 14.5 ± 1.3 mm [23] in the second molar region and 8.7 ± 2.1 mm [23] and 9.9 ± 2.9 mm [22] in the canine area, respectively, as follows:

1. In the image analysis and scientific visualization software (3D Slicer 5.2.1, 3D Slicer), upload the CBCT (RayScan Alpha; RayScan Technologies GmbH; Meersburg, Germany) scan (Add DICOM Data). Select the volume-rendering module from the module list (Modules: Volume Rendering) and select the preset bone visualization (Display: Preset; Select a preset: CT-Bone) option. The volume must be enabled on the volume-rendering module;
2. Set up the Hounsfield scale at a lower value for a better view of the posterior palatal area on the 3D reconstruction by using the specific shift tool (Display: Shift). The value must be set at 150 HU. Change the module from volume rendering to segment editor (Modules: Segmentation Editor). Perform the first segmentation of the maxillary bone by using the “Threshold” tool (Figure 1);

3. Set up the Hounsfield scale value at 1500 HU for a better view of the maxillary teeth. Perform the second segmentation for maxillary teeth. Superimpose the second segmented model with an intraoral scan (IOS), (3Shape TRIOS 3; 3Shape A/S; Copenhagen K, Denmark);
4. Mark the right and left greater palatal artery pathways by using the curve tool on the “Markups to Model” module (Modules: Markups to Model: Curve). Check the coincidence of both the right and the left pathways and anatomical bone landmarks in all section views and in the 3D-reconstruction view (Figure 2);

5. Save all four models in the standard tessellation file (STL) format;
6. In the 3D mesh-processing software system (MeshLab 2022.02; MeshLab), superimpose all four models and the IOS (Figure 3);
7. Save the IOS in the STL format using the system coordinate of obtained models (Figure 3);

8. In the 3D-modeling software (Autodesk Meshmixer 11.5.474; Autodesk Inc.; San Rafael; California; United States of America), upload the IOS and the greater palatal artery models (Figure 4);
9. Align the greater palatal artery pathways with the IOS surface model using the “Align to Target” tool (Select: Edit: Align to Target) (Figure 4);
10. Generate a new model where the mucosal projection of the greater palatal artery pathways can be identified by applying Boolean operations through the IOS model and both greater palatal artery duplicate models. In the guided surgery planning software (BlueSkyPlan 4.12.13; Blue Sky Bio LLC; Libertyville; Illinoise; United States of America), a soft-tissue-free graft splint for a palatal-guided harvesting procedure can be designed (Figure 4);

In the 3D-modeling software (Autodesk Meshmixer; Autodesk Inc.), generate a 3D model with a 3D design for future surgical harvest areas by applying different Boolean operations. This model can be visualized (3Shape 3D Viewer, 3Shape A/S) and sent to a printing machine to obtain a 3D visualization harvesting guide of the FGG depth and position (Figure 5).

This digital technique can easily be replicated for various cases by employing 3D maxillary bone reconstruction based on CBCT. Additionally, it could be adapted to any dental treatment planning software used to design and fabricate a surgical guide for milling or printing.

**Figure 6:**
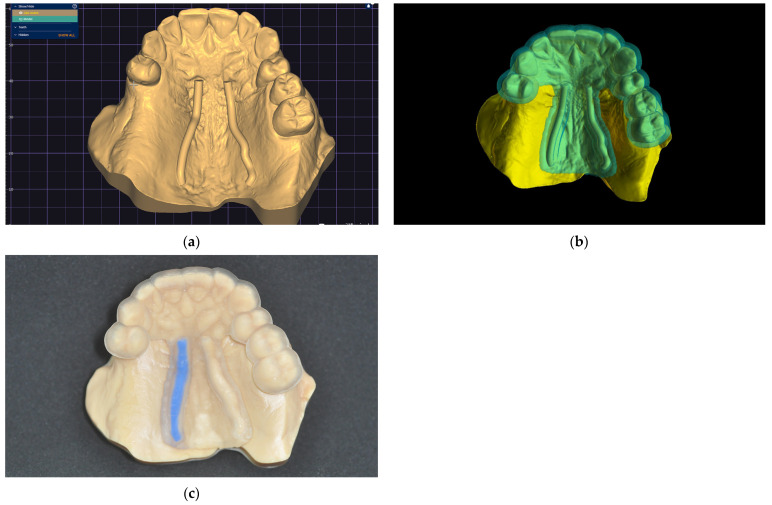
The greater palatal artery pathway surface projection: (**a**) Digital model; (**b**) Digital windowed surgical guide; (**c**) Printed model and static surgical guide with the right greater palatal artery pathway marked on the surface.

A clinical case is presented to illustrate the application of the technique for FGG harvest from the palatal mucosa to increase the quality of keratinized mucosa around lower jaw implants after performing a lower-jaw second right molar extraction (Figure 6). The patient previously had signed a consent form agreeing to the surgical intervention. Following the previously described technique, a digital model was obtained and a digital surgical guide with a windowed safe zone area was designed (BlueSkyPlan; Blue Sky Bio LLC). Both the model (Anycubic Dental Non-Castable Skin UV Resin for LCD Photon 3D printer; Anycubic Photon Mono 4K, Shenzen Anycubic Technology Co., Ltd.; Shenzen; China) and the surgical guide (Anycubic UV Tought Clear Resin for 3D Printing; Shenzen Anycubic Technology Co., Ltd.; Shenzen; China) were printed (3D Anycubic Photon Mono 4K Printer; Shenzen Anycubic Technology Co., Ltd.; Shenzen; China), washed, and cured (Anycubic Wash & Cure Machie 2.0; Shenzen Anycubic Technology Co., Ltd.; Shenzen; China), and the right artery pathway was marked by filling it with light-curing flowable dental composite blue (Colourflow, Dental Life Sciences Ltd., Bucharest, Romania) (Figure 6).

**Figure 7:**
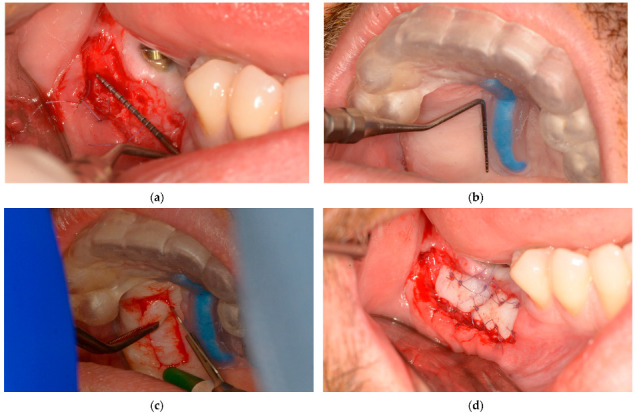
Lower-jaw buccal side wall and upper-jaw donor site measurements and surgical approach: (**a**) The buccal soft-tissue minimum thickness approach; (**b**) Upper jaw with the static windowed surgical guide applied and safe-zone area measurements; (**c**) Upper-jaw FGG harvesting guided by the bordered safe-zone area; (**d**) Buccal side wall with grafted tissue immediately postsurgery.

During the microsurgical approach for soft-tissue augmentation, the thickness of the buccal wall was clinically measured and compared with that of the upper-jaw mucosal-free-graft donor site. This measurement was taken after the surgical guide was placed on the upper jaw, and the mucosal projection of the greater palatal artery was identified (Figure 7). Subsequently, the FGG was properly placed to increase the width of the mandibular keratinized mucosa on the buccal side of the peri-implant tissue, ensuring long-term maintenance (Figure 7).

## 3. Discussion

The technique described here allows for the creation of a static surgical guide with artery pathways marked on its surface, which can be applied to any clinical case through CBCT analysis and surgical planning and design software. The identification of the artery pathway and its precise projection onto the guide surface through software functions offer the surgeon a predictable method for enlarging or restricting the harvesting area of the palatal mucosa and, consequently, to adapt the surgical technique for the receiving area, thereby avoiding intersecting the great palatal artery pathway.

However, precise positioning of the surgical guide could be challenging sometimes, so prior to surgical intervention, the surgical guide could be placed on the upper jaw and the correct positioning checked by the practitioner. Also, a different solution, consisting of using a transparent resin for printing the guide and windowing the buccal or occlusal side of the splint, could be used to properly position the surgical guide on the upper jaw, which would allow the clinician to verify the full insertion on the upper arch. In addition, the use of fixation pins with a guided sleeve could be another alternative method to restrain the uncontrolled movement of the guide during the surgery.

This digital approach for obtaining different types of surgical guides offers multiple possibilities for combining two or more design techniques to create new surgical guides with specific functionalities.

The literature described harvesting techniques for FGGs from the palatal mucosa [11,12,13,14,15] are surgical approaches without digital assistance. They are only based on anatomical landmarks, clinical investigations, and the surgeon’s experience.

The greater palatal artery pathway projection guide offers practitioners a safe area within which to harvest FGGs through digital design and is a time-saving method for permanent control of the intersection of the greater palatal artery pathways. The presence of a surgical guide could extend the safe area for harvesting FGGs based on the distance from the CEJ of the maxillary teeth [22] to the greater palatal artery pathways on both sides of the palatal bone, regardless of artery diameter [21] and depth [23] variations or variable thicknesses of the palatal mucosa [21]. In addition, the separate zones of intervention between the landmarked area (based on the palatal mucosa) and the crestal implant position could be combined using digital design. This approach could allow the use of a single tool for the guided harvesting of FGGs or CTGs and for guided implant positioning.

Despite all precautions to avoid the injury of the palatal vessels, hemorrhages can occur. In the case of type III or IV branching patterns of the greater palatal artery (25% frequency) previously mentioned [20], the greater palatal artery passes very close to the CEJ of maxillary teeth. Different authors [17,18] recommend that the greater palatal artery should be pre-sutured distal to the graft harvesting site before the surgical procedure to limit a possible hemorrhage produced by injuring the artery.

While emphasizing the value of the methods used daily to identify the path of the greater palatal artery, such as CBCT [26], the bony palatal groove containing the entire pathway of the greater palatal artery sometimes cannot be clearly identified. However, using the present technique, the safety zone for palatal grafting incisions could be visualized on the static surgical guide and, consequently, bordered and restrained by the surgeon.

Current protocols for harvesting palatal connective tissue grafts are well standardized. The addition of a guide indicating the course of the palatine artery may be recommended, especially in situations where other guided surgical procedures are indicated, such as for the placement of implants or for other normal or pathological anatomical structures. These can include guiding anesthesia through the palatine foramina, guiding access for periapical surgery, or for the extraction of impacted teeth.

The procedure described is easy to perform and the cost of printing the guide is low, and the benefit–cost ratio justifies its use. Ultimately, the major benefit is the safety measure obtained by the surgeon during the intervention.

The limitation of the study consists in the evaluation being based on a single case, and a larger number of clinical cases are needed in future studies for accuracy testing and to validate the workflow. Also, another limitation of the present technique includes the evaluation by artery tracing, where the bulge in soft tissue remains subjective, and future studies of the technique where the depth of incision is controlled are needed to obtain a higher predictability.

While traditional palatal soft-tissue graft harvesting methods are effective, they are associated with notable complications that can affect patient outcomes. The development and implementation of such novel techniques as described here aim to mitigate these issues, offering improved safety, reduced morbidity, and enhanced patient comfort. A comprehensive review of the literature supports the need for these innovations to address the limitations of conventional methods.

## 4. Conclusions

The described technique offers clinicians a viable method which could be applied for the following purposes:

- Avoiding injury to the greater palatal artery during surgery;
- Visualizing safe areas for FGG harvesting or CTG harvesting.

Finally, static surgical guides incorporating artery pathway projection could achieve the following:

- Aid in intraoperative decision-making;
- Increase surgical predictability.

## Data Availability

The data that support the findings of this study are available from the corresponding author upon reasonable request.

## References

[1] Stimmelmayr M., Allen E.P., Gernet W., Edelhoff D., Beuer F., Schlee M., Iglhaut G. (2011). Treatment of gingival recession in the anterior mandible using the tunnel technique and a combination epithelialized-subepithelial connective tissue graft-a case series. Int. J. Periodontics Restor. Dent..

[2] Mostafa D., Fatima N. (2022). Gingival Recession And Root Coverage Up To Date, A literature Review. Dent. Rev..

[3] Cohen E.S. (1994). Ridge augmentation utilizing the subepithelial connective tissue graft: Case reports. Pract. Periodontics Aesthet. Dent..

[4] Hall H.D., O’ Steen A.N. (1970). Free grafts of palatal mucosa in mandibular vestibuloplasty. J. Oral Surg..

[5] Han T.J., Takei H.H. (1996). Progress in gingival papilla reconstruction. Periodontol. 2000.

[6] Migliorati M., Amorfini L., Signori A., Biavati A.S., Benedicenti S. (2015). Clinical and Aesthetic Outcome with Post-Extractive Implants with or without Soft Tissue Augmentation: A 2-Year Randomized Clinical Trial. Clin. Implant. Dent. Relat. Res..

[7] Zucchelli G., Tavelli L., McGuire M.K., Rasperini G., Feinberg S.E., Wang H., Giannobile W.V. (2020). Autogenous soft tissue grafting for periodontal and peri-implant plastic surgical reconstruction. J. Periodontol..

[8] Tavelli L., Barootchi S., Stefanini M., Zucchelli G., Giannobile W.V., Wang H. (2022). Wound healing dynamics, morbidity, and complications of palatal soft-tissue harvesting. Periodontol. 2000.

[9] van Nimwegen W.G., Raghoebar G.M., Zuiderveld E.G., Jung R.E., Meijer H.J.A., Mühlemann S. (2018). Immediate placement and provisionalization of implants in the aesthetic zone with or without a connective tissue graft: A 1-year randomized controlled trial and volumetric study. Clin. Oral Implant. Res..

[10] Edel A. (1974). Clinical evaluation of free connective tissue grafts used to increase the width of keratinised gingiva. J. Clin. Periodontol..

[11] Langer B., Calagna L.J. (1982). The subepithelial connective tissue graft. A new approach to the enhancement of anterior cosmetics. Int. J. Periodontics Restor. Dent..

[12] Raetzke P.B. (1985). Covering localized areas of root exposure employing the “envelope” technique. J. Periodontol..

[13] Bruno J.F. (1999). A subepithelial connective tissue graft procedure for optimum root coverage. Atlas Oral Maxillofac. Surg. Clin. N. Am..

[14] Hürzeler M.B., Weng D. (1999). A single-incision technique to harvest subepithelial connective tissue grafts from the palate. Int. J. Periodontics Restor. Dent..

[15] Zucchelli G., Mele M., Stefanini M., Mazzotti C., Marzadori M., Montebugnoli L., De Sanctis M. (2010). Patient morbidity and root coverage outcome after subepithelial connective tissue and de-epithelialized grafts: A comparative randomized-controlled clinical trial: Patient morbidity and root coverage outcome after grafts. J. Clin. Periodontol..

[16] Griffin T.J., Cheung W.S., Zavras A.I., Damoulis P.D. (2006). Postoperative complications following gingival augmentation procedures. J. Periodontol..

[17] Greenstein G., Cavallaro J., Tarnow D. (2008). Practical application of anatomy for the dental implant surgeon. J. Periodontol..

[18] Kulkarni M.R., Shettar L.G., Bakshi P.V., Nikhil K. (2021). Palatal pre-suturing for perioperative hemostasis at free gingival graft donor sites: A randomized, controlled clinical trial. J. Periodontol..

[19] Benninger B., Andrews K., Carter W. (2012). Clinical measurements of hard palate and implications for subepithelial connective tissue grafts with suggestions for palatal nomenclature. J. Oral Maxillofac. Surg..

[20] Yu S.K., Lee M.H., Park B.S., Jeon Y.H., Chung Y.Y., Kim H. (2014). Topographical relationship of the greater palatine artery and the palatal spine. Significance for periodontal surgery. J. Clin. Periodontol..

[21] Ki D.H., Won S.Y., Bae J.H., Jung U.W., Park D.S., Kim H.J., Hu K. (2014). Topography of the greater palatine artery and the palatal vault for various types of periodontal plastic surgery. Clin. Anat..

[22] Tavelli L., Barootchi S., Ravidà A., Oh T.J., Wang H.L. (2019). What Is the Safety Zone for Palatal Soft Tissue Graft Harvesting Based on the Locations of the Greater Palatine Artery and Foramen? A Systematic Review. J. Oral Maxillofac. Surg..

[23] Herman L., Font K., Soldatos N., Chandrasekaran S., Powell C. (2022). The Surgical Anatomy of the Greater Palatine Artery: A Human Cadaver Study. Int. J. Periodontics Restor. Dent..

[24] Chan H.L., Wang H.L., Fowlkes J.B., Giannobile W.V., Kripfgans O.D. (2017). Non-ionizing real-time ultrasonography in implant and oral surgery: A feasibility study. Clin. Oral Implants Res..

[25] Hilgenfeld T., Kästel T., Heil A., Rammelsberg P., Heiland S., Bendszus M., Schwindling F.S. (2018). High-resolution dental magnetic resonance imaging for planning palatal graft surgery-a clinical pilot study. J. Clin. Periodontol..

[26] Miwa Y., Asaumi R., Kawai T., Maeda Y., Sato I. (2018). Morphological observation and CBCT of the bony canal structure of the groove and the location of blood vessels and nerves in the palatine of elderly human cadavers. Surg. Radiol. Anat..

[27] Velloso G., Zimmermann D., Shibli J.A., Dias A.T., Moraschini V. (2023). A multifunctional guided surgery to assist in the 3-dimensional positioning of dental implants and in obtaining a palatal gingival graft. J. Prosthet. Dent..

[28] Chen Z., Li J., Sinjab K., Mendonca G., Yu H., Wang H.L. (2018). Accuracy of flapless immediate implant placement in anterior maxilla using computer-assisted versus freehand surgery: A cadaver study. Clin. Oral Implants Res..

